# Brief Interventions for Families Seeking Support from Family Services: A Scoping Review

**DOI:** 10.3390/ijerph22060841

**Published:** 2025-05-27

**Authors:** Victoria Hamilton, Gina-Maree Sartore, Michelle Macvean, Elbina Avdagic, Zvezdana Petrovic, Cathryn Hunter, Catherine Wade

**Affiliations:** Parenting Research Centre, 696 Bourke Street, Melbourne, VIC 3000, Australia; gsartore@parentingrc.org.au (G.-M.S.); mmacvean@parentingrc.org.au (M.M.); eavdagic@parentingrc.org.au (E.A.); zpetrovic@parentingrc.org.au (Z.P.); chunter@parentingrc.org.au (C.H.); cwade@parentingrc.org.au (C.W.)

**Keywords:** brief interventions, family services, vulnerable families, scoping review

## Abstract

Brief family support interventions may be an effective and acceptable option when demands on services and pressures on families can often mean intensive, long-term family support interventions are an inefficient and unappealing course of action. The purpose of this scoping review was to better understand the nature of non-medical brief interventions targeted at parents and families experiencing adversity or challenging circumstances that may lead them to seek support from family services. We used a systematic search and selection process to identify publications (papers or webpages) about brief interventions for parents and families within three academic databases and 70 websites. Publications were in scope if the interventions were targeted to parents and families, were non-medical in nature, and were brief (no longer than 10 h duration, or up to four sessions). We identified 78 papers and webpages eligible for inclusion in this scoping review, covering 46 brief interventions. Data were extracted by two researchers and charted in a spreadsheet. Most interventions were delivered in the mental health sector, followed by the education, and then community or family services sector, and most often in a clinical setting. Intervention duration varied, ranging from 45 min to a two-day workshop, and were usually aimed at improving the mental health of children and young people. Interventions were delivered to groups of parents, followed by whole families or individual parents. This review highlights the pressing need for high-quality evaluations of brief interventions for family support, and given the diversity of delivery modes, durations and conceptualisation of ‘brief intervention’ in the field and literature, further synthesis of the evidence through systematic reviews is required. This paper advances understanding and clarity on how brief interventions may be beneficial for families experiencing adversity, yet further evaluation and systematic review for acceptability and efficacy is required.

## 1. Introduction

Families seeking support services can be offered a range of options, some of which will involve brief interventions to provide support and inform parents quickly and efficiently. Brief interventions are typically time-limited, content-limited and use specific strategies to help clients alleviate immediate needs or to initiate interactions between client and professionals. Brief interventions may also be referred to as ‘simple advice’, ‘minimal interventions’, ‘brief counselling’ or ‘short-term counselling’ [1] (p. 3). Brief interventions can be used stand-alone or in conjunction with other interventions and generally address specific problems or goals rather than larger concerns [1]. With the focus on a specific objective and client-centredness, brief interventions can provide a quick avenue for building client relationships [1] and may also have a social benefit associated with increasing client engagement with support. This may lend brief interventions to services being able to provide support to families in a prompt and efficient way, overriding some of the practical challenges and delays sometimes associated with traditional interventions offering longer-term comprehensive treatment approaches.

There is no universally agreed definition of what constitutes a brief intervention [2]. Generally, brief interventions appear not to exceed four or five sessions, although the optimal number of sessions is unclear [3]. A systematic review in the area of childhood anxiety treatment operationalised an intervention as ‘brief’ if it included at least 50% fewer total sessions than standard treatment, as ‘intensive’ if the number of sessions and intervention duration is reduced versus standard treatment (e.g., one 180 min session treatment for specific phobia), and ‘concentrated’ if the intervention has a standard number of sessions, but sessions are delivered in a shorter time period (e.g., 12 sessions of CBT delivered in 6 weeks) [4]. Brief interventions have also reportedly been as brief as a 30 s behavioural change intervention to more than 30 min for an extended brief behavioural intervention [5]. In a systematic review of universal family and child health services, a duration limit of four sessions was within the review scope [2].

There is systematic review evidence to suggest brief interventions may result in improved child outcomes; however, the scope of these reviews is largely focused on populations with mental health concerns [6,7,8,9,10,11,12]. While this provides some indication of suitability and effectiveness of brief interventions within the mental health setting, these reviews provide limited understanding of the applicability of brief interventions for the diverse range of families, in addition to those with mental health concerns, that may engage with family support services. A better understanding of the nature of brief interventions for a broader range of populations would support an understanding of the potential for this type of intervention to be effectively adopted to meet the needs of families seeking support. To address the gap in research, this scoping review aims to examine the purpose, populations, settings, dose and delivery of brief interventions for parents and families that are likely to attend family support services. While there is currently no evidence indicating the level of demand for brief interventions across family services, there is widespread implementation and uptake of this modality. With brief interventions increasingly adopted across family services to provide more immediate support, this review offers a unique opportunity to describe brief intervention approaches in family support services.

## 2. Method

### 2.1. Review Design

We conducted a scoping review to develop a picture of brief interventions used with families. We drew on the scoping review framework developed by Arksey and O’Malley [13] and refined by Levac and colleagues [14] for mapping out the nature of and evidence for brief interventions for families needing support, which has not been extensively reviewed. This guided our approach to conducting the scoping review, including identification of the research question, the relevant literature, publication selection, publication charting, and the collation, summary and reporting of publications. While we adopted the Preferred Reporting Items for Systematic Reviews and Meta-Analyses (PRISMA-ScR), the checklist was not registered.

### 2.2. Eligibility Criteria

Any study design reporting parent, child, family or service outcomes was in scope. Searches within databases were limited to the English language and the year 2018 onwards to capture recent research, but no year limits were applied to webpage searches. Brief interventions were in scope if they were synchronous and targeted parents or families experiencing adversity, including the following: child maltreatment; housing instability or being priced out of stable housing; mental health concerns; emotional or behavioural concerns; under- or unemployment; family violence; community violence; disability; alcohol or other drug misuse; eating disorders; incarceration; school attendance concerns; sleep and settling concerns; and attachment issues. Interventions delivered to children without their parents involved, or interventions aimed at adults who were not identified as parents, were excluded. Interventions relating to medical concerns, such as cancer or diabetes, were ineligible. The interventions could be delivered by professionals, paraprofessionals, peers, or volunteers in group, one-to-one, or dyad (e.g., couple, parent–child) formats. Delivery could be in-person, via telephone or teleconference, text-based, or web-based.

As there is no uniform duration for brief interventions in the literature, we took the approach of including interventions of up to four sessions [2] or up to 10 h duration [15], including single-session interventions. Interventions were excluded from the review if duration in sessions or hours was not reported.

### 2.3. Information Sources

We included published and unpublished studies, webpages, practice guides, and programme manuals that provided some detail regarding brief interventions for parents or families. Only online sources were included, while books, book chapters and theses were excluded. We selected bibliographic databases and search engines suitable for scoping reviews in the social sciences field [16], and conducted a comprehensive search across PsychInfo, Cumulative Index for Nursing and Allied Health (CINAHL), SocINDEX on 3 May 2023 and 70 websites in May 2023 (see Appendix A for a list of organisation websites searched). We also invited our colleagues with expertise in family support services to suggest potential studies and brief interventions for consideration in this review.

### 2.4. Search Strategy

We developed a priori search terms designed to identify various types of brief interventions, including single-session and walk-in approaches, brief workshops, and casual and low-intensity programmes (see Appendix B). Search terms were also used to identify interventions specifically for parents, caregivers and families. Truncation symbols were used to identify all possible variations of the key terms with the Boolean ‘OR’ criterion, with the search initially designed to identify individual terms. Combinations of the key terms were subsequently searched using the Boolean ‘AND’ criterion.

### 2.5. Selection and Extraction

Publications identified through database and website searches and by expert recommendation were imported into Endnote bibliographic software, Version 21 to support our screening processes. Duplicates were removed and then titles and abstracts (or website excerpts where applicable) were screened against pre-defined inclusion and exclusion criteria. Full text was obtained for all publications that were screened in, and these were subsequently reviewed by three researchers from the review team (EA, CH and ZP). Uncertainties as to eligibility were resolved by consultation with the full review team.

### 2.6. Data Charting

A data charting spreadsheet was developed to guide and chart the systematic extraction of information from all included publications. The extraction categories included intervention name, target outcome, country, service sector, target population, intervention duration/dose, setting, structure, mode and a summary of the intervention. Three members of the review team (EA, CH and ZP) extracted key details from full text publications into spreadsheet categories and collaborated with the full review team to resolve any ambiguous decisions by consensus.

## 3. Results

### 3.1. Search and Selection Results

**Figure 1:**
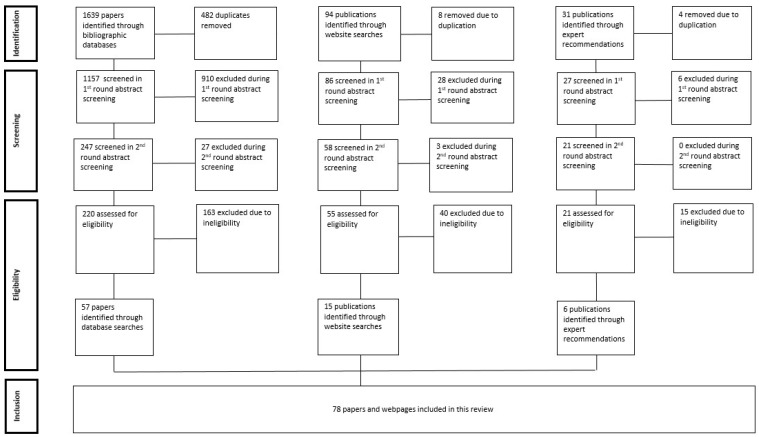
PRISMA flow diagram of the search and selection process.

An initial 1764 publications (inclusive of articles and webpages) were identified through database and website searches and through expert recommendations. After the initial screening process, 296 publications were assessed in detail for final eligibility, with a total of 78 publications retained. This included 57 papers from bibliographic databases, 15 websites, and 6 publications through expert recommendations. The strategy for study selection is depicted in Figure 1, which outlines how the PRISMA guidelines for inclusion of studies was applied. Study characteristics, intervention and outcomes are summarised in Table 1.

### 3.2. Service and Setting Contexts for Brief Interventions

From the 78 publications examined in this review, brief interventions were predominantly reported in the mental health sector (*n* = 24), the education sector (*n* = 15) and community or family services sectors (*n* = 11). We found 10 publications reporting interventions in the health sector, primarily in maternal health and antenatal care. Two interventions were delivered in the disability sector, and one each in child protection and corrections. One publication was not sector specific.

Interventions were primarily delivered in a clinical setting, such as a hospital, therapeutic clinic or treatment centre (*n* = 29). Interventions delivered in universities (*n* = 10), community service agencies (*n* = 7), the home (*n* = 4) and community settings (*n* = 5) were also reported. A further six were delivered online through teleconferencing and four were delivered in schools.

### 3.3. Purpose and Domains of Brief Interventions

Most brief interventions aimed to improve the mental health of children and young people. Some publications reported interventions targeting mental health and psychological symptoms broadly (*n* = 14), while others named specific child mental health conditions such as anxiety (*n* = 6), depression (*n* = 3) and suicidal thoughts and behaviours (*n* = 1). Some interventions targeted externalising behaviours of children and young people, including child behaviour in general (*n* = 8), and use of alcohol and other drugs (*n* = 5). Others were intended to improve child outcomes such as eating disorders (*n* = 2), sleep issues *(n* = 3), executive functioning (*n* = 1) and overall development (*n* = 1).

Some interventions targeted parent mental health including stress, distress, worry (*n* = 4), general mental health (*n* = 2) and conditions such as anxiety (*n* = 3) and depression (*n* = 3). Parenting, including positive parenting, parenting competence and dysfunctional parenting (*n* = 8) were also the focus of several interventions. Various interventions aimed to build specific parenting skills in the areas of parenting self-efficacy (*n* = 7), parent knowledge (*n* = 2), self-esteem (*n* = 1), parent psychological flexibility *(n* = 2), parent attitudes (*n* = 1), reflective functioning (*n* = 1) and mentalisation (*n* = 2).

Some publications described brief interventions targeting family-level outcomes including family worry and confidence (*n* = 1), family conflict (*n* = 2) and family functioning (*n* = 2), or improving family and/or parent–child relationships *(n* = 9). One paper targeted increasing joint attention in a parent–child dyad. Brief interventions were also used to motivate and enhance engagement of families and children in further interventions (*n* = 5).

### 3.4. Families Participating in Brief Interventions

The populations targeted in most interventions were people with mental health concerns (child mental health = 23 publications, parental mental health = 7, parent distress = 4). Children with disabilities were the target population in 13 publications, including children with Down’s Syndrome, Attention Deficit Hyperactivity Disorder and Autistic children. People with alcohol and other drug concerns were the target population of eight interventions, and three sources described interventions aimed broadly at vulnerable or at-risk populations, while others targeted low income (*n* = 3) and families priced out of housing (*n* = 1). Some interventions targeted families experiencing conflict or intimate partner or family violence (*n* = 4) and one targeted refugees of war. One intervention was aimed at pregnant adolescents, three targeted children and young people with eating disorders, and three interventions were for children with sleep disorders.

Many interventions targeted broad age ranges from young children to early adolescence. Thirteen interventions targeted families of children aged from 7 to 14 years, or 4 to 15 years, while three interventions targeted families of children aged 0 to 18 years. However, some interventions had narrow child age groups, specifically for adolescents, usually stating 12 to 19 years (*n* = 9), newborns and infants (*n* = 3) or toddler and preschool years (*n* = 3). There were also four publications on brief interventions delivered during the antenatal period.

### 3.5. Duration of Brief Interventions

There was considerable variation in the duration of brief interventions within the scope of our selection, which was limited to interventions delivered over a maximum of four sessions or up to 10 h duration. Several interventions consisted of one session only (*n* = 16) which ranged from 45 min to a two-day workshop. A further 16 interventions consisted of one primary session with an add-on, which was either a refresher session, text message to reinforce content, an individual family-member session or coaching phone calls. The remaining interventions involved two to four core sessions of widely varying duration.

Single-session approaches were adopted by several brief interventions identified in this review. Single-session intervention (SSI) is an approach to therapy and service delivery which promotes a client-centred and collaborative approach when working with individuals, couples and families. In the literature, it is referred to as Single Session Therapy, Single Session Work and Single Session Thinking. While there are some interventions that use the name single-session, these may be one-off encounters with the client (e.g., a workshop) that do not follow the SSI approach. SSI is underpinned by research and clinical findings suggesting many clients seeking therapy only attend one session and significantly benefit from it [81]. This does not mean that SSI is restricted to one session, but that each session, particularly the first one, is viewed as potentially the final session. Thus, the aim of each session is to maximise therapeutic benefits for the client while leaving an opportunity for having additional sessions, if required. The typical structure of SSI involves having one session which is followed up with a phone call a few weeks later to ascertain how the client is going and whether further support is required. A decision whether to have an additional session(s) is based on discussions between the practitioner and the client [82].

### 3.6. Delivery of Brief Interventions

Various delivery structures were identified, sometimes with multiple modes used in the one intervention. Interventions were most often delivered to groups of parents (*n* = 22), the whole family, or multiple family members (*n* = 15), individual parents (*n* = 15), groups of couples (*n* = 3), an individual couple (*n* = 1), individual parent–child dyads (*n* = 5), groups of parent–child dyads (*n* = 1), whole family plus teacher (*n* = 1), parent/family session plus child session (*n* = 3), whole family session plus child session (*n* = 1), and parent session plus parent–child session (*n* = 1). These were a mix of individual and group level interventions. There were also some interventions that used various delivery structures depending on which family members opted to attend any given session (*n* = 7).

The intervention delivery approaches identified more often in the review included psychoeducation, motivational interviewing, parent coaching and cognitive behavioural therapy, with some delivering a manualised, structured approach and others tailoring sessions to suit client-identified needs or goals.

## 4. Discussion

The purpose of this scoping review was to map the breadth and nature of the literature describing brief interventions for families experiencing circumstances that lead them to seek formalised support. Within this population, a wide range of brief interventions were identified with diverse applications, dosage, targeted outcomes and delivery approaches. This review indicates brief interventions in the provision of family support are implemented internationally and predominantly in the mental health field, targeting the mental health of children and young people. Brief interventions are also widely implemented across community or family services, education, maternal health and antenatal care, disability, child protection and corrections. However, there may be limits to the severity of challenges that can be addressed with brief interventions. While not examined in the current review, there is concordance in the literature that brief interventions are suitable for mild to moderate difficulties and are typically considered to be low intensity [83]. Some authors go so far as to suggest [84] that brief interventions will not help clients with complex or acute needs.

The brief interventions described in publications identified in the current review ranged from well-known, researched, manualised and prescribed programmes, to novel innovations and flexible, tailored approaches with room to expand the amount and type of assistance according to family needs and interests. There was considerable variation in how much intervention families received, and how interventions were delivered, including interventions that combined individual with group work, didactic learning with self-directed learning, online with face-to-face delivery, and family-level supports with parent-level supports. Clinical and therapeutic approaches were employed in several of the interventions, sometimes using more than one approach in the same intervention. Consideration should be given to the suitability of brief interventions where there is a high risk to health and safety. In these cases, some triaging by services may be warranted to determine suitability for brief interventions. Given the wide variation in intervention delivery approaches, there is no clear indication in the literature as to an ideal brief intervention approach. The ideal intervention approach is likely to be governed by a range of factors, including family context, and the severity of the presenting issues.

While most brief interventions included in this review focused on addressing mental health concerns in clinical, mental health settings, there were some examples of use in broader populations and settings. For example, several brief interventions targeted families experiencing a diverse range of concerns or vulnerabilities, or in community and school settings. Thus, in addition to the mental health sector, there are some indications of the applicability of brief interventions to the broader family services sector. The interventions in this review typically involved groups of parents attending an intervention, and often one parent only or in some cases multiple family members, but infrequently involved couples, parent–child dyads or parallel sessions with children and younger people alongside parents. The focus on parents as the agent of change in the brief interventions reported here is consistent with the parenting support approach taken in many family services, suggesting brief interventions have further applicability in this context.

In services where there is potential for a moderate or high number of families to attend once or infrequently, single-session interventions may be a viable option. Some common elements of SSIs include identifying the client’s goals, checking periodically whether the session is on track, providing feedback and focusing on responding to the client’s goals [85]. In many cases, these elements could be sufficient to address immediate family needs, while remaining open to further support provision or onward referral. Single-session approaches therefore have the potential to capitalise on an initial interaction to provide support, in what may be the only opportunity to address families’ goals. Such an approach promotes the likelihood that families walk away from a service with something helpful, regardless of whether they return in future. As often is the circumstance of vulnerable families, their concerns, needs and challenges may come and go or resurface over time [86]. In SSIs, intervention does not cease when all challenges are resolved, but instead the end of an intervention signals that the client is better able to self-manage their current concern. When considering ‘how much is enough’ of an intervention, the duration is less significant than whether the intervention has been sufficient in helping the client address their immediate needs [86].

Another advantage to the single-session way of working is that it can be viewed as a philosophical approach rather than a specific intervention or programme, as there is no prescribed type of therapy within SSI and practitioners can use their own therapeutic modality while delivering the session in a way that is consistent with single-session thinking [85]. This flexibility allows agencies and practitioners to employ practices that suit them and their clients within the single-session model. Several publications included in this scoping review noted the benefits of SSIs for supporting engagement as an approach that could be used with the intention of providing immediate assistance or supporting motivation to attend a further service. The objective of a single session could entail providing families with as much knowledge and relevant strategies and skills as possible, as early as possible. Several of the interventions included in this scoping review appeared to adopt an approach congruent with the SSI model, such as when families participate in an initial session or two involving motivational interviewing to support engagement, and this is then followed up with feedback and coaching to support continued change.

Based on the literature, the single-session philosophy shares some similar features with solution-focused therapy, as both methods focus less on identifying problems and concerns and more on what can be implemented to achieve an immediate solution to a goal, possibly drawing on past successes and strengths. This has been identified by others as one of the favourable qualities of SSIs when used to support First Nations people [70], who underline the value of the oral tradition and deep listening in therapeutic conversation with Indigenous families when using the single session approach. Other potential strengths of a single-session approach include the focus on clients’ needs and resources rather than on intake processes and assessment completion. Meeting people where they are at and letting clients take the lead in the conversations were also reported advantages [70]. Practices seen to support a good experience with single-session interventions included deep listening, respecting the oral tradition, not using jargon but instead using the language of the family and not approaching the session as an expert [70].

### Limitations of This Review

We imposed several pragmatic limits to our search process (e.g., English language papers only, a definition of ‘brief intervention’, and publications from 2018 onwards) which may have meant we missed key papers or websites of relevance to the aims of this review. Additionally, our databases were predominantly in the fields of health and mental health, and there may be additional perspectives from other applied social sciences lacking in this review. Broader search parameters in future reviews may contribute to furthering understanding of what works and for whom, beyond the conclusion drawn from our review.

## 5. Conclusions

Brief interventions appear to sufficiently address the immediate needs of families seeking formal support and may be particularly useful for enhancing motivation and engagement with supports. Their application may, however, be better suited to clients with low- to moderate-level needs or risks.

While the publications included in this review suggest brief interventions that have more frequently been used in the context of mental health services, there are also examples of use in family support services with families experiencing a range of challenges. Brief interventions, particularly those with single-session approaches, may complement the range of approaches available in family support services. Given the potential for variations in definitions of brief intervention, any policies recommending the use of brief interventions should ideally articulate how brief interventions are to be conceptualised, in terms of duration, scope, purpose and delivery. Routine evaluation of brief interventions is particularly important given the variability in what is conceptualised as a brief intervention, and when new approaches are designed or adapted.

## Figures and Tables

**Table 1:** Characteristics, interventions and outcomes of included publications.

Intervention	Target Populations	Target Outcomes	Service Sectors	Settings	Modalities Used Duration Range
1-2-3 Magic [17]	Parents of children aged 6–12 years diagnosed with ADHD	Reduction in child disruptive behaviour, ADHD symptoms, dysfunctional parenting	Mental health	University	3 × 2 h weekly sessions
ACORN [18]	Pregnant women with elevated anxiety symptoms	Reduction in parental anxiety	Prenatal/pregnancy health	Antenatal/parenting classes	3 × 2 h group sessions for pregnant women and their partners
Bedtime Fading for Preschoolers [19]	Children 1.5 years old with sleep difficulties and their mothers	Child sleep onset latency Child wake after sleep onset Child bedtime tantrums	Family services	University	2 × 90 min sessions with groups of parents
Brief Acceptance and Commitment-based interventions [20,21,22,23,24]	Mothers with infants who screen positive for illicit substance use Mothers of autistic children 0–22 years Parents of autistic children 5–13 years Parents who have experienced relationship violence	Substance use treatment uptake Parental depression, stress, social isolation and physical health Positive parenting practices	Health Community Mental health	Hospital Community School	1–3 × 45 min sessions with individual parents 1.5 day workshop with groups of caregivers followed by a refresher session 2 × 2 h sessions with groups of parents 1 evening session, followed by a 1 full-day session and a refresher session with groups of parents 4 × 1 h sessions with group of parents
Brief cognitive behavioural group intervention for parents of anxious children [25]	Parents of primary school children aged 4–10 years, who have reported their child is experiencing symptoms of anxiety without a formal diagnosis	Reduction in child anxiety symptoms Increases in parent self-efficacy	Community	Community: Intended for delivery in schools but due to COVID-19 lockdowns it was delivered online	3 online sessions with groups of parents (2 × 2 h; 1 × 1.5 h) delivered fortnightly
Brief Relationship Intervention and Screening [26,27]	Children 0–17 years who have experienced family violence and their non-offending parent/caregiver	Child emotional behavioural difficulties and traumatic stress response Parental psychological distress, reflective function, sensitivity and responsiveness Positive parent–child interactions Parenting self-efficacy Positive attitudes to help seeking	Community	Community	4 × 60–90 min sessions with individual parents and child–parent dyad
Brief Training Program for Primigravid Adolescents [28]	Married primigravid adolescents	Parenting self-efficacy Mother-infant bonding Social support	Health	University	3 × 60–90 min sessions with groups of pregnant women
Coping Effectiveness Training (CET) [29]	Caregivers of autistic children	Coping self-efficacy Feasibility of the CET intervention	Health	Clinic	2 sessions with individual parents (1 × 90 min and 1 × 60 min)
Creating Connections [30]	Mothers of newborns (1–14 days old)	Feasibility of a technology-based intervention	Paediatric	Ambulatory paediatric centre and home	10–15 min plus text messages over 4 months
Early Screening for Therapy and Empowering Parents (STEPS) [31]	Caregivers and children 0–6 years	Parenting satisfaction Uptake of referrals and follow-on services	Paediatrics/Health	Paediatric clinic	1 × 45 min sessions with individual parents and children
Emotion-Focused Therapy Workshop [32,33,34,35]	Parents of children, young people and other family members with mental health concerns	Parenting self-efficacy Child behaviour Family psychological symptoms Family engagementChild mental health difficulties	Mental health	Clinic Online	2-day workshop with groups of parents
Family Check-up [36,37,38]	Families with children or young people with conduct or behavioural concerns; low income families.	Child substance use Child mental health Child behaviour Family relationships Peer relationships	Health Community Menmintal health	Hospital School Community Clinic	1–3 × 45 min sessions with individual parents Between 1 and 2 sessions or a 1.5 day workshop with groups of parents
Family Minds [39,40]	Foster parents with at least one foster or adopted child who was 4 years or older	Increased parental reflective functioning Reduction in parenting stress	Foster care Child protection	Foster care Child protection	3 × 3 h classes over 4–6 weeks with groups of foster carers
Family Model [41]	Children <18 years experiencing mental illness and a parent/caregiver experiencing mental illness	Development of family-focused care plan	Hospital Child and Youth Mental Health Service	Clinic	1 × 1–2 h individual session with child and their family
Fear-less Triple P [42,43,44]	Parents/caregivers of children 6–14 years with anxiety	Child anxiety Sibling anxiety Parental confidence Child emotion-regulation strategies	Community	Online	1 × 2 h seminar with group of parents 1 × day (8 h) or 3 days (2.5 h) workshop with groups of parents
Focused parent-infant psychotherapy [45]	Depressed mother with an infant with sleep disorder	Infant regulatory behaviours Parenting competence	Family services	Clinic	4 sessions with individual infant-parent dyad
Get a Grip on anxiety [46]	Parents/caregivers of children 7–12 years old with anxiety	Child anxiety and depression	University	University Home	2 × 2 h workshops with groups of parents 10 × 1 h weekly programme with individual parent and child
Home Base Program [47]	Parents/caregivers and their adolescent children aged 12–17 years who use substances	Decreased alcohol use and increased family cohesion	University	University Home or other location for parent delivered sessions Phone for coaching support	1 × 4 h parent training 3 × sessions delivered by parents to their adolescents3 × “coaching” phone calls (15–20 min) prior to each parent–child session
Home Based Adolescent Sexual Education for intellectual Disabilities [48]	Parents of individuals 12–30 years old with Down Syndrome	Parental attitudes surrounding sexuality and disability Parental self-efficacy in discussing sexuality	Intellectual disability	Clinic	3 × 3 h workshops with groups of parents
HUGS Mother-infant Interaction Intervention [49]	Mothers diagnosed with depression and their infants 0–12 months old	Mother-infant relationship Maternal parenting stress Early child development	Mental health	Clinic	4 × 1.5 h sessions with groups of parent–child dyads
Integrated Alcohol and Suicide Intervention for Suicidal Teens [50]	Suicidal adolescents (13–18 years) and their caregivers	Adolescent alcohol consumption Adolescent suicidal thoughts and behaviours	Mental health	Inpatient psychiatry unit	1 × 60–90 min individual sessions with adolescents1 × 20–30 min family sessions
Joint attention training [51]	Caregivers of autistic children (3–6 years old)	Improve joint attention	University	University	2–3 × 10 min sessions per week with a parent–child dyad
Let’s Talk About Children (Let’s Talk) [52,53]	Parents experiencing mental illness	Parenting Family functioning	Mental health	Mental health service	2 × 1 h sessions with individual parents (preferably both parents) with possible additional 3rd session if more complex issues are present
Newborn Behavioural Observations [54]	First time mothers with antenatal distress and at risk of postnatal depression and their babies	Mother-infant interaction quality Maternal anxiety or depression symptoms Depression diagnosis	Healthcare/maternal healthcare	Hospital Home	3 × 20–40 min sessions
One-session group based parenting intervention [55]	Parents with anxiety disorders and their children (3–9 years old)	Child anxiety	Mental health	NHS/University	1 × 5.5 h interactive workshop
One Session Psycho-educational Workshop [56]	Parents/caregivers of children 4–15 years at risk of ADHD or who have been diagnosed with ADHD	Parental beliefs and knowledge about ADHD Treatment acceptance and utilisation	Community	School district office University	1 × 2 h workshop with groups of parents
Parent Coaching [57]	Parents who have already attended parenting courses	Parental knowledge in attachment Parent–child relationship Parental confidence	Family services	Wellbeing and Family Relationship service Online	Up to 3 sessions with individual parents
Positive Family Holistic Health Intervention [58]	Children and young people (13 years and older) on probation and their families	Physical activity and fitness performance Self-esteem Happiness Anxiety and depression symptoms Life satisfaction Quality of life Family communication and wellbeing Relationship with probation officers	Probation service	Community	2 × 1 h sessions with individual clients 1 × 4.5 h activity with groups of families
Positive Parenting Intervention [59]	Mothers with history of depression and their children aged 8–10 years	Positive parenting behaviours Child positive affect	Mental health	University	1 × 90 min session with individual parents and parent–child dyads
Primary Care Stepping Stones Triple P and Stepping Stones Triple P Seminars [60,61]	Parents of children with a disability aged 2–12 years	Child behaviours Child developmental issues	Community	School Child care centres	Stepping Stones Triple P Seminar Series—1–3 × 90 min sessions with groups of parents Primary Care Stepping Stones Triple P—4 × 15–20 min individual parent sessions
RAD-PAL intervention (adaptation of Teen Intervene) [62]	Adolescents (13–17 years) who use substances and their parents/caregivers	Adolescent substance use	University	Community	1 × parent session and 2 × adolescent sessions
Ready? Set. Go! [63]	Children 3–5 years who experience homelessness and high mobility and their parents	Child executive function	Community	Homelessness shelter Community preschool	3 × 2 h sessions with groups of parents
Reducing Family Conflict Discussion Group [64]	Parents of adolescents 11–16 years where there is a concern about parent-adolescent conflict	Family conflict Adolescent Behaviour Problems Parenting and parent-adolescent relationship	Community	Community	1 × 2 h sessions with groups of parents
Relationship Education [65]	Pregnant women on low income	Relationship distress and satisfaction Parental stress and coping	Community	Community	4 × 3 h sessions with groups of expectant mothers and their partners
School readiness coaching [66]	Parents of children (3–5 years old) attending a well-child clinic	Parents’ perception of school readiness coaching intervention	Paediatrics	Paediatric clinic	1 h session with a mother and a child (15 min child assessment and 45 min parent coaching)
Sexuality Training [67]	Parents/caregivers of children diagnosed with intellectual and developmental disabilities	Parental attitudes and beliefs, level of communication and competence to teach sexuality topics	Education and disability	Autism and developmental disorder conference	1 × 60 min session with groups of parents
Short-term Psychodynamic Infant-Parent Intervention at Child Health Centres [68]	Mothers of children 0–2 years who are experiencing distress	Parental stress Infant socio-emotional functioning	Mental health Child health centres	Child health centres	4 × 45 min sessions with individual parents or individual parent–child dyads or individual whole families
SOS-DoC framework counselling sessions [69]	Pregnant women who experienced intimate partner violence	Family function (family communication, family support, family difficulty)	Antenatal	Hospital/Antenatal clinic	3 × 1–2 h individual sessions at two-week intervals
Single-session interventions * [70] Single Session Family Consultation [71] Single Session Family Therapy [72] Walk-In Together Online Session [73]	Aboriginal families participating in therapy Children and young people with mental health difficulties and their families Children and young people with mental health difficulties and their families Families experiencing various vulnerabilities or adversities	Family-identified goals Family engagement Family-identified goals Family worry Family confidence Family-identified goals	Mental health Mental health Community	Clinic Clinic Community Online	1–3 × 1–1.5 h sessions with the family and a follow-up phone call 1 or more sessions with the family and a follow up phone call 1 × 60–75 min session with at least two family members
Single Session Intervention [74]	Families with children with eating disorders (who are on a waitlist for Family Based Therapy)	Child eating disorder	Counselling and treatment service	Clinic and online	1 × up to 90 min individual session
Single-session parent sleep educational intervention [75]	Parents of school-aged children (7–14 years) with neurodevelopmental or mental health disorders and with a suspicion of a behavioural sleep-problem	Child sleep	Mental health	Clinic	1 × 45 min session with groups of parents
Single Session Personalised Intervention [76]	War refugee parents of adolescents 10–15 years [76]	Parental self-efficacy	University	Home	1 × session with individual parents
Solution-focused Brief Therapy [77]	Families referred for family therapy	Family-identified goals	Family services	Community	1 × 68 min session with family
Uncle Lightfoot [78]	Parents of children 3–8 years with nighttime fears	Child nighttime fears	University	Home	1 training session with individual parents Each evening × 5 weeks parent delivered therapy (6 hrs per week on average)
Video-feedback intervention [79]	Mother with depressive symptoms and her baby	Therapeutic change expression during a mentalisation-informed intervention	MH	Online	1 pre-intervention session and 4 intervention sessions with mother-child dyad
Workshop for Family Based Treatment [80]	Parents of adolescents with restrictive eating disorders	Parental self-efficacy Adolescent weight gain	Health	Specialist service	1 × 3 h workshop with groups of parents

* Single-session interventions using a Single Session Therapy/Single Session Work/Single Session Thinking approach where each session is viewed as possibly the last one while providing an open door for having more sessions, if required.

## Appendix A

**Table A1:** Organisation websites searched.

Organisation	Website
Act for Kids	https://www.actforkids.com.au accessed on 2 May 2023
Allright	https://www.allright.org.nz/tools/parenting-courses accessed on 10 May 2023
Anglicare Australia	https://www.anglicare.asn.au accessed on 9 May 2023
Anglicare NSW & ACT	https://www.anglicare.com.au/ accessed on 9 May 2023
Anglicare NT	https://www.anglicare-nt.org.au/ accessed on 9 May 2023
Anglicare QLD	https://anglicarecq.org.au/ accessed on 9 May 2023https://www.anglicanchurchsq.org.au/anglicare accessed on 4 May 2023https://www.anglicarenq.org.au/ accessed on 4 May 2023
Anglicare SA	https://anglicaresa.com.au/ accessed on 4 May 2023
Anglicare Tasmania	https://www.anglicare-tas.org.au/ accessed on 9 May 2023
Anglicare Victoria	https://www.anglicarevic.org.au/ accessed on 4 May 2023
Anglicare WA	https://www.anglicarewa.org.au/ accessed on 9 May 2023
Australian Childhood Foundation	https://www.childhood.org.au accessed on 4 May 2023
Australian Indigenous HealthInfoNet	https://healthinfonet.ecu.edu.au accessed on 3 May 2023
Australian Institute of Aboriginal and Torres Strait Islander Studies	https://aiatsis.gov.au/research accessed on 3 May 2023
Australian Institute of Family Studies	https://aifs.gov.au accessed on 3 May 2023
Australian Institute of Health and Welfare	https://www.aihw.gov.au accessed on 3 May 2023
Berry Street	https://www.berrystreet.org.au/ accessed on 2 May 2023
beyondblue	https://www.beyondblue.org.au accessed on 4 May 2023
Black Dog Institute	https://www.blackdoginstitute.org.au accessed on 4 May 2023
BlueKnot Foundation	https://blueknot.org.au accessed on 4 May 2023
Body Matters Australia	https://bodymatters.com.au accessed on 4 May 2023
Bouverie Centre	https://www.latrobe.edu.au/research/centres/health/bouverie accessed on 2 May 2023
Child and Adolescent Mental Health Service (CAMHS) ACT	https://www.canberrahealthservices.act.gov.au/services-and-clinics/services/child-and-adolescent-mental-health-service-camhs-community-teams accessed on 4 May 2023
CAMHS NSW	https://www.health.nsw.gov.au/mentalhealth/Pages/services-camhs.aspx accessed on 4 May 2023
CAMHS SA	https://www.wchn.sa.gov.au/our-network/camhs accessed on 4 May 2023
CAMHS Tasmania	https://www.health.tas.gov.au/health-topics/mental-health/tasmanias-mental-health-system/child-and-adolescent-mental-health-service accessed on 4 May 2023
CAMHS WA	https://cahs.health.wa.gov.au/Our-services/Mental-Health accessed on 4 May 2023
Canadian Child Welfare Research Portal	https://cwrp.ca accessed on 10 May 2023
CatholicCare ACT	https://catholiccare.cg.org.au/ accessed on 9 May 2023
CatholicCare Central QLD	https://catholiccarecq.com/ accessed on 9 May 2023
CatholicCare NSW	https://www.catholiccare.org accessed on 9 May 2023
CentaCare SA	https://www.cccsa.org.au/ accessed on 9 May 2023
CatholicCare Tasmania	https://catholiccaretas.org.au/ accessed on 9 May 2023
CatholicCare Victoria	https://www.catholiccarevic.org.au/ accessed on 9 May 2023
Centre for Excellence in Child and Family Welfare	https://www.cfecfw.asn.au accessed on 10 May 2023
Centre for Integrative Health	https://cfih.com.au accessed on 4 May 2023
Child Welfare Information Gateway	https://www.childwelfare.gov accessed on 10 May 2023
Communicare	https://www.communicare.org.au/Children-Youth-Family/Parenting-Services/Parenting-Support-Services accessed on 4 May 2023
COPE	https://www.cope.org.au accessed on 4 May 2023
Child and Youth Mental Health Service (CYMHS) QLD	https://www.childrens.health.qld.gov.au/services/mental-health accessed on 4 May 2023
CYMHS Victoria	https://www.alfredhealth.org.au/services/child-youth-mental-health-service accessed on 4 May 2023https://www.easternhealth.org.au/mental-health-3/infants-children-and-youth-0-25/ accessed on 4 May 2023
Drummond Street Services	https://ds.org.au/ accessed on 2 May 2023
Family Life	https://www.familylife.com.au accessed on 2 May 2023
ForWhen	https://forwhenhelpline.org.au accessed on 4 May 2023
Headspace	https://headspace.org.au/, Accessed on 2 May 2023
Health Navigator NZ	https://www.healthnavigator.org.nz/healthy-living/p/parenting-resources-courses-and-support/ accessed on 10 May 2023
JewishCare NSW	https://jewishcare.com.au accessed on 9 May 2023
JewishCare QLD	http://www.jcareqld.com/ accessed on 9 May 2023
JewishCare Victoria	https://www.jewishcare.org.au/ accessed on 9 May 2023
Lowitja Institute	https://www.lowitja.org.au accessed on 3 May 2023
MacKillop Family Services	https://www.mackillop.org.au accessed on 3 May 2023
Meerlinga	https://www.meerilinga.org.au/parenting-courses-services/ accessed on 4 May 2023
Mental Health Beacon	https://www.latrobe.edu.au/__data/assets/pdf_file/0006/1153959/Mental-Health-Beacon-Project-Report-March-2015.pdf accessed on 4 May 2023
National Society for the Prevention of Child Cruelty	https://www.nspcc.org.uk/, Accessed on 10 May 2023
Ngala	https://www.ngala.com.au accessed on 9 May 2023
Orygen	https://www.orygen.org.au accessed on 3 May 2023
PANDA	https://panda.org.au/ accessed on 4 May 2023
Relationship Australia	https://relationships.org.au accessed on 3 May 2023
SNAICC	https://www.snaicc.org.au accessed on 3 May 2023
Social Care Institute for Excellence	https://www.scie.org.uk accessed on 10 May 2023
Turning Point	https://www.turningpoint.org.au accessed on 4 May 2023
Uniting SA	https://unitingsa.com.au/ accessed on 10 May 2023
Uniting WA	https://unitingwa.org.au/ accessed on 9 May 2023
UnitingCare Australia	https://unitingcare.org.au, Accessed on 9 May 2023
Uniting NSW & ACT	https://www.uniting.org/home, Accessed on 9 May 2023
UnitingCare QLD	https://www.unitingcareqld.com.au/ accessed on 9 May 2023
Uniting Victoria & Tasmania	https://www.unitingvictas.org.au/services/ accessed on 9 May 2023
Wanslea	https://www.wanslea.org.au/families-and-children/parents-and-grandparent-carers#Parenting-support accessed on 9 May 2023

## Appendix B

**Table A2:** Search Terms.

1	Brief N3 (educat* or interven* or therap* or counsel* or facilit* or program* or help* or assist* or guid* or navigat* or helpline* or support* or advisor* or advice or consult* or helpline* or practice* or coach* or interviewing)
2	Walk-in N3 (educat* or interven* or therap* or counsel* or facilit* or program* or help* or assist* or guid* or navigat* or helpline* or support* or advisor* or advice or consult* or helpline* or practice* or coach* or interviewing)
3	Drop-in N3 (educat* or interven* or therap* or counsel* or facilit* or program* or help* or assist* or guid* or navigat* or helpline* or support* or advisor* or advice or consult* or helpline* or practice* or coach* or interviewing)
4	Short N3 (educat* or interven* or therap* or counsel* or facilit* or program* or help* or assist* or guid* or navigat* or helpline* or support* or advisor* or advice or consult* or helpline* or practice* or coach* or interviewing)
5	Casual N3 (educat* or interven* or therap* or counsel* or facilit* or program* or help* or assist* or guid* or navigat* or helpline* or support* or advisor* or advice or consult* or helpline* or practice* or coach* or interviewing)
6	Low-intensity N3 (educat* or interven* or therap* or counsel* or facilit* or program* or help* or assist* or guid* or navigat* or helpline* or support* or advisor* or advice or consult* or helpline* or practice* or coach* or interviewing)
7	Workshop* N3 (educat* or interven* or therap* or counsel* or facilit* or program* or help* or assist* or guid* or navigat* or helpline* or support* or advisor* or advice or consult* or helpline* or practice* or coach* or interviewing)
8	Single-session N3 (educat* or interven* or therap* or counsel* or facilit* or program* or help* or assist* or guid* or navigat* or helpline* or support* or advisor* or advice or consult* or helpline* or practice* or coach* or interviewing)
9	One-off N3 (educat* or interven* or therap* or counsel* or facilit* or program* or help* or assist* or guid* or navigat* or helpline* or support* or advisor* or advice or consult* or helpline* or practice* or coach* or interviewing)
10	One-session N3 (educat* or interven* or therap* or counsel* or facilit* or program* or help* or assist* or guid* or navigat* or helpline* or support* or advisor* or advice or consult* or helpline* or practice* or coach* or interviewing)
11	Light-touch N3 (educat* or interven* or therap* or counsel* or facilit* or program* or help* or assist* or guid* or navigat* or helpline* or support* or advisor* or advice or consult* or helpline* or practice* or coach* or interviewing)
12	Parent* or carer* or caregiver* or care-giver* or mother* or father* or family or families or mum* or dad* or mom* or maternal or paternal or stepparent* or guardian* or kin or kith or mob*
13	1–11/or
14	12 and 13

* In a Boolean search the asterisk is a truncation to identify variations of a word.
